# Integrating Pulmonary Health and Maternal Care: Bridging Gaps for Optimal Respiratory Outcomes in Pregnancy and Beyond

**DOI:** 10.7759/cureus.64794

**Published:** 2024-07-18

**Authors:** Vasiliki E Georgakopoulou, Chrysoula Taskou, Antigoni Sarantaki

**Affiliations:** 1 Department of Pathophysiology/Pulmonology, Laiko General Hospital, Athens, GRC; 2 Department of Midwifery, Faculty of Health and Caring Sciences, University of West Attica, Athens, GRC

**Keywords:** interdisciplinary collaboration, asthma management, pregnancy, maternal care, pulmonary health

## Abstract

The integration of pulmonary health and maternal care is critical for ensuring optimal respiratory outcomes for both mothers and their infants. Pregnancy induces significant physiological changes in the respiratory system, increasing the risk of pulmonary complications and exacerbating conditions such as asthma. This editorial emphasizes the necessity for collaborative care between pulmonologists and midwives to manage these challenges effectively. By working together, healthcare providers can develop comprehensive care plans that address potential respiratory issues early, monitor and manage chronic conditions, and provide vigilant postpartum care. Enhanced education and interdisciplinary training for both professions are essential for bridging the gaps in care and improving maternal and neonatal health outcomes. This integrated approach is supported by research demonstrating the benefits of coordinated care models in reducing complications and promoting better health outcomes.

## Editorial

Pulmonary complications during pregnancy, such as asthma exacerbations, can significantly impact both maternal and fetal health. Asthma is one of the most common chronic conditions affecting pregnant women, with approximately 8% of pregnant women suffering from it [1]. Poorly controlled asthma can lead to adverse pregnancy outcomes, including preeclampsia, preterm birth, and low birth weight [1]. Therefore, it is essential for pulmonologists to work closely with midwives to monitor and manage asthma effectively during pregnancy. This collaboration ensures that both the mother and the developing fetus receive appropriate care.

The physiological changes in the respiratory system during pregnancy, including increased oxygen consumption and decreased lung capacity, can exacerbate existing pulmonary conditions or unmask previously undiagnosed issues. These changes necessitate a proactive approach to monitoring respiratory health. Pulmonologists are well-equipped to understand these complex changes, while midwives are often the first point of contact for pregnant women. By integrating their expertise, healthcare providers can develop comprehensive care plans that address potential respiratory issues early on, thus preventing complications [2].

Moreover, the postpartum period presents additional challenges for respiratory health. Conditions such as postpartum hemorrhage and infections can impact lung function, requiring vigilant monitoring and management. Midwives play a crucial role during this period, providing continuous care and support to new mothers. Collaboration with pulmonologists can enhance this care, ensuring that any respiratory issues are promptly identified and treated. This integrated approach can also facilitate better management of chronic conditions like asthma, reducing the risk of long-term complications for both the mother and the child [3].

Education and training are key components in bridging the gap between pulmonologists and midwives. By incorporating respiratory health education into midwifery training programs, midwives can be better prepared to identify and manage pulmonary issues. Similarly, pulmonologists can benefit from understanding the unique needs and challenges of pregnant and postpartum women. Joint training sessions and interdisciplinary workshops can foster mutual understanding and cooperation, ultimately improving patient outcomes [4].

Research supports the need for integrated care models to improve maternal and neonatal health outcomes. Studies have shown that coordinated care involving multiple specialties leads to better management of chronic conditions and reduces the incidence of complications [5]. Implementing such models requires commitment from both pulmonologists and midwives, as well as support from healthcare institutions. By working together, these professionals can ensure that pregnant women receive the comprehensive care they need, promoting better health for both mother and child.

In conclusion, the integration of pulmonary health and maternal care is crucial for optimal respiratory outcomes in pregnancy and beyond. Collaborative efforts between pulmonologists and midwives can significantly improve the management of conditions like asthma, enhance monitoring of respiratory health during pregnancy, and provide comprehensive postpartum care. By fostering a culture of cooperation and mutual respect, healthcare providers can bridge the gaps in maternal and pulmonary care, ensuring the best possible outcomes for mothers and their babies.
